# Economic Implications of Dementia in the United States: Assessing the Relationship Between Direct Medical Spending and Informal Care Costs

**DOI:** 10.1002/alz.087727

**Published:** 2025-01-09

**Authors:** Amy Lastuka, Michael Breshock, Joseph L Dieleman

**Affiliations:** ^1^ Institute for Health Metrics and Evaluation, Seattle, WA USA

## Abstract

**Background:**

There are 6.7 million people living with dementia in the United States, and this number is projected to increase as the population ages. The cost of informal care makes up a large proportion of the societal costs of dementia due to the significant care needs associated with the condition.

**Method:**

We combined previously created estimates of direct medical spending attributable to dementia with estimates of informal care costs to arrive at a total societal cost for dementia. Informal care estimates are based on survey data from the Health and Retirement Study, the National Health and Aging Trends Study, and the Behavioral Risk Factor Surveillance System. Both replacement cost and forgone wage cost are estimated for informal care.

**Result:**

We estimated that the annual societal cost attributable to dementia in 2019 was $60,311 (95% uncertainty interval [UI] $56,230–$64,507) per prevalent case when informal case is measured in terms of replacement cost, and $36,245 (95% UI $34,562–$37,960) per prevalent case when informal care cost is measured in terms forgone wages of caregivers. The contribution of informal care to total costs varied substantially by state, ranging from 58% (95% CI 55‐60%) in Connecticut to 84% (95% CI 83‐85%) in New Mexico when measured in terms of replacement cost. When measured in terms of forgone wages, the contribution of informal care ranged from 43% (95% CI 41‐45%) in Connecticut to 69% (95% CI 67‐70%) in Arizona.

We found a negative relationship between direct and indirect costs of dementia. When disaggregating based on type of care, we found that this inverse relationship was driven by long‐term care and ambulatory care. The cost of informal care was not correlated with the cost of emergency department care or inpatient care.

**Conclusion:**

The inverse relationship between direct and indirect costs of dementia provides suggestive evidence that they act as substitutes. While the substitution between informal care and long‐term care is unsurprising, more research is needed to understand potential mechanisms linking informal care and ambulatory care costs.

